# Comparative metabolic responses and adaptive strategies of wheat (*Triticum aestivum*) to salt and alkali stress

**DOI:** 10.1186/s12870-015-0546-x

**Published:** 2015-07-07

**Authors:** Rui Guo, Zongze Yang, Feng Li, Changrong Yan, Xiuli Zhong, Qi Liu, Xu Xia, Haoru Li, Long Zhao

**Affiliations:** Institute of Environment and Sustainable Development in Agriculture (IEDA), Chinese Academy of Agricultural Sciences (CAAS)/Key Laboratory of Dryland Agriculture, Ministry of Agriculture, Beijing, 100081 P.R. China; Key laboratory of Molecular Epigenetics of Ministry of Education (MOE), Northeast Normal University, Changchun, 130024 China

**Keywords:** Wheat, Salt stress, Alkali stress, Growth, Photosynthesis, Metal elements, Free ions, Metabolites

## Abstract

**Background:**

It is well known that salinization (high-pH) has been considered as a major environmental threat to agricultural systems. The aim of this study was to investigate the differences between salt stress and alkali stress in metabolic profiles and nutrient accumulation of wheat; these parameters were also evaluated to determine the physiological adaptive mechanisms by which wheat tolerates alkali stress.

**Results:**

The harmful effect of alkali stress on the growth and photosynthesis of wheat were stronger than those of salt stress. High-pH of alkali stress induced the most of phosphate and metal ions to precipitate; as a result, the availability of nutrients significantly declined. Under alkali stress, Ca sharply increased in roots, however, it decreased under salt stress. In addition, we detected the 75 metabolites that were different among the treatments according to GC-MS analysis, including organic acids, amino acids, sugars/polyols and others. The metabolic data showed salt stress and alkali stress caused different metabolic shifts; alkali stress has a stronger injurious effect on the distribution and accumulation of metabolites than salt stress. These outcomes correspond to specific detrimental effects of a highly pH environment.

**Conclusions:**

Ca had a significant positive correlation with alkali tolerates, and increasing Ca concentration can immediately trigger *SOS* Na exclusion system and reduce the Na injury. Salt stress caused metabolic shifts toward gluconeogenesis with increased sugars to avoid osmotic stress; energy in roots and active synthesis in leaves were needed by wheat to develop salt tolerance. Alkali stress (at high pH) significantly inhibited photosynthetic rate; thus, sugar production was reduced, N metabolism was limited, amino acid production was reduced, and glycolysis was inhibited.

**Electronic supplementary material:**

The online version of this article (doi:10.1186/s12870-015-0546-x) contains supplementary material, which is available to authorized users.

## Background

Salinization has been considered as a major environmental threat to agricultural systems; approximately 20 % of arable land and 50 % of irrigated land worldwide have been affected by salinity [1, 2]. K^+^, Na^+^, Ca^2+^, Mg^2+^, Cl^−^, NO_3_^−^, HCO_3_^−^, CO_3_^2−^, and SO_4_^2−^ are predominant ions in natural saline soils. Läuchli and Lüttge confirmed that soil salinization and alkalization occur simultaneously [3]. Thus far, studies have mainly focused on salt stress [4, 5]. Although studies on high-pH calcareous soils, alkaline soils, and alkaline salt stress have also conducted, studies on alkali stress have been rarely performed [6–8].

Salt stress induces osmotic stress and ion injury by disrupting ion homeostasis and ion balance in plant cells; alkali stress exhibits the same stress factors but becomes aggravated when combined with high-pH stress [9–12]. A highly alkaline environment in the rhizosphere can reduce mineral element availability by precipitating Ca^2+^, Mg^2+^, and HPO_3_^−^; as a result, ion uptake is inhibited and ion homeostasis is disrupted [13, 14]. High pH can also immediately destroy root membrane structure and strongly affect structural functions, such as break intracellular ion balances [15]. Thus, plants in alkaline soil must cope with physiological drought and ion toxicity, maintain intracellular ion balance, and regulate pH outside roots. Plant responses to alkali stress may involve metabolic pathways, such as ion transport, photosynthesis, osmotic solute accumulation, and hormone synthesis. Metabolomic solutes, such as proline, betaine, polyamine, and polyhydric alcohol, contribute to salt stress tolerance. Metabolomic components may participate in plant alkali tolerance; however, information regarding alkali tolerance-related metabolomic components is limited. A comparative metabolic analysis of plant response to salt and alkali stress will be conducted to determine metabolomic components with high pH-specific response. These metabolomic components are also necessary to understand plant alkali tolerance. Metabolomic analyses have been applied in functional genomic research, which can reveal specific responses of biological systems to genetic and environmental changes [16]. Metabolomic analyses have been performed to investigate mechanisms related to salt stress adaptation and tolerance, such as ion homeostasis, osmotic stress, and detoxification [1]. Practically, metabonomics involves detecting and quantifying the metabolic changes with techniques such as gas chromatography-mass spectrometry (GC-MS), liquid chromatography-Fourier transform mass spectrometry (LC − FT/MS), and nuclear magnetic resonance (NMR) [17]. These technologies can be employed to accurately identify metabolomic components [18–20].

In this study, wheat seedlings were treated with salt stress (9:1 molar ratio of NaCl:Na_2_SO_4_) or alkali stress (9:1 molar ratio of NaHCO_3_:Na_2_CO_3_). We systematically analyzed the metabolomic features of wheat and their dynamic responses to salt and alkali stresses using GC–MS technology in conjunction with multivariate data analysis [21]. The objectives of this study are to further define the metabolomic of wheat plants and determine the physiological adaptive mechanisms by which wheat tolerates alkali stress.

## Methods

### Plant materials and growing conditions

The seeds of wheat (*T. aestivum*) cv Dan-4589, a salt-resistant cultivar, were sown in 34 cm diameter plastic pots containing 5.5 kg of washed sand. Each pot contained five seedlings. The pots were watered daily with 0.5× Hoagland nutrient solution at 17:00 to 18:00. All of the pots were maintained in a greenhouse at 22.5 ± 1.5 °C at daytime and at 18.5 ± 1.5 °C at nighttime. The plants grew at uniform irradiance of photosynthetic photon flux density of 300 μmol m^−2^ s^−1^.

### Stress treatments

Twenty pots with seedlings growing uniformly were selected and divided randomly into four sets when the seedlings were four weeks old; each set comprised five pots. Each pot was considered a single replicate, with five replicates per set. One set was used as untreated control group, a second set was used to determine the growth index at the beginning of treatment, and the last two sets were used as stress treatment groups. Salt stress was simulated by mixing neutral salts (NaCl and Na_2_SO_4_) at a molar ratio of 9:1 and applying 80 mM of salt mixture (pH 6.86, EC 8690 μs/cm). Alkali stress was simulated by mixing NaHCO_3_ and Na_2_CO_3_ at a molar ratio of 9:1 and applying at 80 mM of the mixture (pH 9.08, EC 6550 μs/cm). The pots subjected to stress treatments were watered daily with the nutrient solution containing salt mixtures at 17:00 to18:00 for 15 d; the control plants were watered with nutrient solution only.

### Measurement of growth and photosynthesis indices

Relative growth rate (RGR) is defined as [ln DW at the end of stress treatment − ln DW at the start of stress treatment]/total treatment duration [22]. The photosynthetic indices were determined at 10:00 from first fully expanded leaf blades by using a LI-6400XT portable open flow gas exchange system (Li-Cor, USA). The plants were treated with photosynthetically active radiation (PAR) of 1000 μmol m^−2^ s^−1^ (saturation irradiance) by using red–blue light-emitting diodes. The photosynthetic pigments were determined in accordance with previously described methods [23]. Chlorophyll (Chl) *a* and *b* and carotenoid (Car) contents were extracted with acetone; each sample was spectrophotometrically analyzed at 440, 645, and 663 nm five times in accordance with previously described methods [24].

### Measurement of metal elements and free ions

Wheat leaves and roots were digested with HNO_3_ by microwave digestion. Na, K, Ca, Mg, Fe, Cu, Zn, and Mn contents were determined using an atomic absorption spectrophotometer (TAS-990, Purkinje General, Beijing). After water extraction was performed, the quantities of the free ions (NO_3_^−^, Cl^−^, SO_4_^2−^, and H_2_PO_4_^−^) were determined through ion exchange chromatography (DX-300 ion chromatographic system; AS4A-SC ion-exchange column, and CD M-II electrical conductivity detector; DIONEX, Sunnyvale, USA) with a mobile phase of 1.7 mM/1.8 mM Na_2_CO_3_/NaHCO_3_ [13].

### Measurement of metabolites

Leaf extract was prepared using via the following procedures. Approximately 100 mg of each frozen tissue sample was transferred into 2 ml centrifuge tubes, and 60 μl of water containing ribitol as an internal standard was added to each tube. After the mixtures were vortexed with 0.3 ml of methanol and 0.1 ml of chloroform, a 70 Hz grinding mill system (Jinxin Biotech LTD. Shanghai, China) was used to grind the samples for 5 min; the samples were then incubated at 70 °C for 10 min. The tubes were centrifuged at 12,000 rpm at 4 °C for 10 min. Supernatant (0.35 ml) was decanted into a 2 ml volume screw-top glass tube; the samples were dried in a vacuum concentrator at 30 °C for 2 h. Afterward, each sample was dissolved in 80 μl of methoxamine hydrochloride and incubated at 37 °C for 2 h. The samples were further derivatized with N,O-bis(trimethylsilyl)-trifluoroacetamide (BSTFA) containing 1 % trimethylchlorosilane (TMCS) (100 μl) at 70 °C for 1 h [25].

GC-TOF/MS analysis was performed using a 1D Agilent 7890 gas chromatograph system coupled with a Pegasus 4D time-of-flight mass spectrometer. The system was equipped with a DB-5MS capillary column coated in 5 % diphenyl cross-linked with 95 % dimethylpolysiloxane (30 m × 250 μm inner diameter and 0.25 μm film thickness; J&W Scientific, Folsom, CA, USA). An aliquot of the analyte (1 μL) was injected in a splitless mode. Helium was used as carrier gas, the front inlet purge flow was 3 mL min^−1^, and the gas flow rate through the column was 1 mL min^−1^. The initial temperature was maintained at 90 °C for 0.25 min; temperature was increased to 180 °C at a rate of 10 °C min^−1^ and to 240 °C at a rate of 5 °C min^−1^. Temperature was further increased to 285 °C at a rate of 20 °C min^−1^ for 11.5 min. Injection, transfer line, and ion source temperatures were 280, 270, and 220 °C, respectively. Energy was set at −70 eV in an electron impact mode. Mass spectrometry data were acquired in a full-scan mode with an *m*/*z* range of 20 to 600 at a rate of 100 spectra per second after a solvent delay of 492 s.

Chroma TOF 4.3X software of LECO Corporation and LECO-Fiehn Rtx5 database were used for raw peaks exacting, the data baselines filtering and calibration of the baseline, peak alignment, deconvolution analysis, peak identification and integration of the peak area [26]. Using LECO’s terminology, the data processing method should have 'Baseline', 'Peak Find', 'Caculate Area/Height' function activated. Baseline offset was set at 1 (just above the noise), data points to be averaged for smoothing was set at automatic, peak width was set at 6s and the maximum number of unknown peaks to find was set to 10000 to get as more features as possible. A signal/noise (S/N) threshold of 50:1 was used [27]. LECO-Fiehn Rtx5 library was used for searching, and the masses 85 through 600 were searched. The minimum similarity before named was assigned was set at 200 to get as more features as possible. Unique masses were used to area/height calculation.

### Statistical analysis

Growth, photosynthetic activity, and inorganic ion variance and correlation were statistically analyzed using SPSS 13.0. All of the treatments were replicated five times; means and calculated standard errors (S.E.) were reported. Metabolites were identified by searching FiehnLib (GC-TOF), a commercial EI-MS library [26]. The resulting three-dimensional data, including peak number, sample name, and normalized peak area, were run in SIMCA 13.0 software package (Umetrics, Umea, Sweden) and subjected to principal component analysis (PCA) and orthogonal projections to latent structure-discriminant analysis (OPLS-DA). Non-commercial databases, including KEGG (http://www.genome.jp/kegg/), were utilized to search for metabolite pathways. Format data were uploaded in the MetaboAnalyst website (www.metaboanalyst.ca/) for further analysis [28].

## Results

### Growth, Photosynthesis, and Pigment contents

**Fig. 1:**
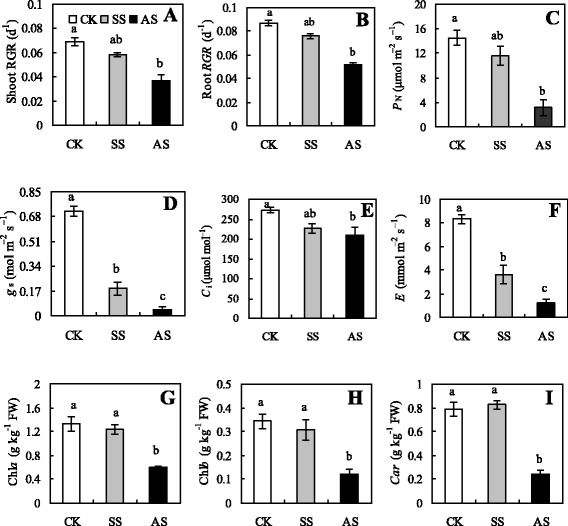
Effects of salt stress (SS) and alkali stress (AS) on relative growth rate (*RGR*) of leaves (**a**) and roots (**b**), net photosynthetic rate (*P*_N_) (**c**), stomatal conductance (*g*_s_) (**d**), intercellular CO_2_ concentration (*C*_i_) (**e**), transpiration rate (*E*) (**f**), chlorophyll *a* (Chl *a*) (**g**) and *b* (Chl *b*) contents (**h**), and carotenoid (*Car*) (**i**) content of wheat. Values are means of five replicates. Means followed by different letters in the same stress type are significantly different at *p* < 0.05 according to Duncan’s method

The growth of wheat seedling leaves and roots decreased under salinity stress compared with that of the control group; the growth of these parts was reduced to a greater extent under alkali stress than under salt stress (Figs. 1a and b, *p* < 0.05). The photosynthetic indices of plants exposed to salt and alkali stress were lower than those of the control plants; however, these parameters decreased sharply under alkali stress (Figs. 1c–f, *p* < 0.05). Pigment contents were not significantly affected by salt stress, but Chl and Car contents decreased remarkably in plants under alkali stress compared with those in the control plants (Figs. 1g–i, *p* < 0.05).

### Metal element and free ion contents

**Fig. 2:**
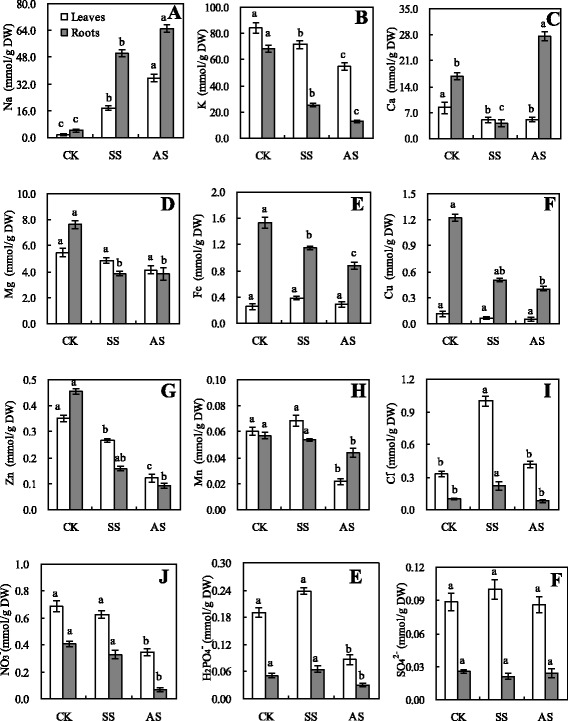
Effects of salt stress (SS) and alkali stress (AS) on the contents of metal elements and free ions in wheat seedling leaves and roots. Metal elements Na (**a**), K (**b**), Ca (**c**), Mg (**d**), Fe (**e**), Cu (**f**), Zn (**g**), and Mn (**h**); free ions Cl^−^ (**i**), NO_3_^−^ (**j**), H_2_PO_4_^−^ (**k**), and SO_4_^2−^ (**l**). Values are means of five replicates. Means followed by different letters in the same stress type are significantly different at *p* < 0.05 according to Duncan’s method

In response to salt and alkali stress for 15 d, the Na content of leaves and roots increased, whereas K content decreased; nevertheless, the magnitude of these changes was greater under alkali stress than under salt stress (Figs. 2a and b, *p* < 0.05). Ca content in leaves was reduced under both stress conditions. Ca content decreased under salt stress, whereas Ca content in roots increased under alkali stress (Fig. 2c, *p* < 0.05). Mg content in leaves slightly changed, whereas Mg content in roots significantly decreased under salt and alkali stress (Fig. 2d, *p* < 0.05). Fe and Cu accumulation in leaves was not affected by both stress conditions compared with that in the control plants (Figs. 2e and f, *p* < 0.05). By contrast, Fe and Cu contents decreased in roots; this decrease was significantly greater under alkali stress than under salt stress (Figs. 2e and f, *p* < 0.05). Zn content in wheat seedlings decreased under salt and alkali stress, although the decrease under alkali stress was significantly lower than that under salt stress (Fig. 2g, *p* < 0.05). Mn content was not significantly affected by salt stress; by contrast, Mn content significantly decrease in leaves and roots, as induced by alkali stress (Fig. 2h, *p* < 0.05). Cl^−^ content was slightly affected by alkali stress; conversely, Cl^−^ content was significantly increased by salt stress (Fig. 2i, *p* < 0.05). NO_3_^−^ and H_2_PO_4_^−^ contents were also not significantly affected by salt stress; NO_3_^−^ and H_2_PO_4_^−^ contents were decreased dramatically by alkali stress (Figs. 2j and k, *p* < 0.05). SO_4_^2−^ content was not affected by salt and alkali stress (Fig. 2l, *p* < 0.05).

### Metabolic changes in response to control, salt stress, and alkali stress treatments

**Fig. 3:**
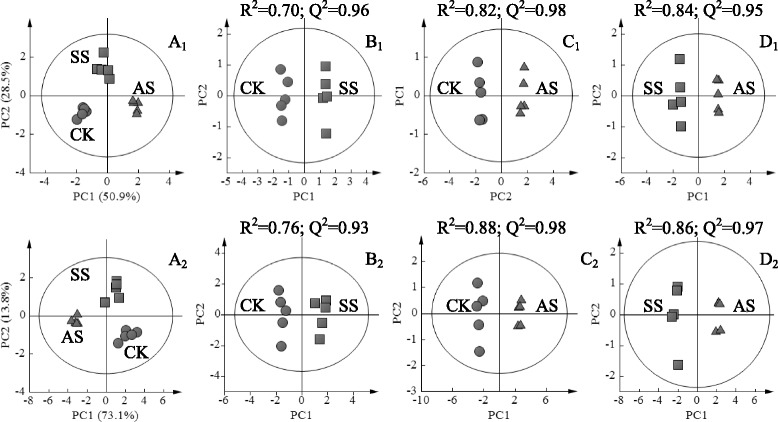
Principal component analysis (PCA) score plots showing the metabolomic trajectory of leaves (A_1_) and roots (A_2_) of wheat seedlings under no salinity stress (CK), salt stress (SS), and alkali stress (AS). Orthogonal partial least squares discriminant analysis (OPLS-DA) scores showing dose dependence of salinity effects on wheat seedlings: CK vs. SS in leaves (B_1_) and roots (B_2_). CK vs. AS in leaves (C_1_) and roots (C_2_). SS vs. AS in leaves (D_1_) and roots (D_2_)

The metabolic changes in leaves and roots of wheat seedlings subjected to control, salt stress, and alkali stress treatments were determined through GC–MS to reveal the physiological responses and adaptive strategies of wheat to salinity stress. A significant difference exists in the metabolite profiles between samples under control and salinity stress treatments. A total of 75 metabolites were identified and determined in leaves and roots. Based on the PCA results, a separation of samples under control treatment, salt stress, and alkali stress in leaves and roots (Fig. 3 and Additional files 1 and 2) was observed. The control and salinity treatment samples in leaves and roots were separated by the first principal component (PC1), representing 50.9 % and 73.1 % of the total variation (Figs. 3a and b). Pairwise comparative OPLS-DA was conducted with one orthogonal and one predictive component calculated for all models derived from two classes of samples to obtain detailed information on metabolic alterations under salt and alkali stress and significance of metabolites contributing to the alterations. In this research, OPLS-DA models revealed the separation between samples within control and salinity treatments. The score plots of OPLS-DA results showed clear separation between wheat plants under salt and alkali stress and control plants with good model quality (Fig. 3 B_1_ and C_1_; B_2_ and C_2_). These differences were also observed in the score plots between salt stress and alkali stress in leaves and roots, respectively (Figs. 3 D_1_ and 3 D_2_).

### Metabolic profiles in response to salt and alkali stress in wheat seedlings

**Fig. 4:**
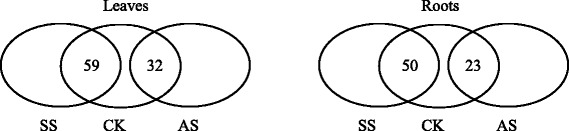
Global comparison of metabolic profiles in leaves and roots under no salinity stress (CK), salt stress (SS), and alkali stress (AS). A total of 77 metabolites were identified in this study, and the numbers in the figure indicate the numbers of metabolites with no significant difference in their contents for each comparison

Based on the results of PCA and OPLS-DA, the responses of metabolites to salt stress and alkali stress were different in leaves and roots. The response of 11 and 5 metabolites under salt stress and 7 and 36 metabolites under alkali stress remarkably increased and decreased, respectively, in leaves of wheat seedlings (Fig. 4, leaves). Following pre-stress, salt stress caused an increase in levels of glucose, glucose-6-P, fructose-6-P, 3-PGA, and PEP, which are involved in glycolysis, and in levels of fructose, trehalose, proline, valine, isoleucine, and leucine. By contrast, salt stress resulted in a decrease in levels of fumaric acid and malic acid, which are involved in the TCA cycle, and in levels of maltose, shikimic acid, and quinic acid, which are involved in the shikimate pathway. Under alkali stress, the levels of some amino acids and sugars increased, including proline, lysine, sucrose, sorbitol, trehalose, lyxose, and gentiobiose; however, glycolysis was significantly inhibited under alkali stress, thereby decreasing the levels of glucose-6-P, fructose-6-P, and PEP. Furthermore, the TCA cycle, which is associated with glycolysis pathway, was inhibited under alkali stress, as shown by lower citric acid, α-ketoglutaric acid, and fumaric acid levels than the control plants. Shikimate pathway and GABA shunt metabolites were inhibited under alkali stress, resulting in a decrease in shikimic acid, quinic acid, phenylalanine, GABA, and glutamate levels (Table 1).

In roots, the response of 9 and 16 metabolites under salt stress and 8 and 44 metabolites under alkali stress significantly increased and decreased, respectively (Fig. 4, roots). Salt stress caused a significant increase in levels of proline, sucrose, myo-inositol, xylitol, galactinol, raffinose, gentiobiose, galactose, phosphate, and diglycerol compared with the control treatment; by contrast, levels of glucose and glucose-6-P, which are involved in glycolysis, some amino acids, such as GABA, valine, and asparagine, and some sugars, including sorbitol, xylose, and lyxose, decreased remarkably in roots. Alkali stress significantly affected the TCA cycle; citric acid, aconitic acid, α-ketoglutaric acid, succinic acid, and fumaric acid were significantly increased, indicating that energy production in the TCA cycle was enhanced by alkali stress. Furthermore, levels of shikimic acid, quinic acid, and diglycerol were significantly increased. Most of the amino acids and sugars in roots decreased under alkali stress compared with those of the control treatment, although this decrease was significantly lower than that under salt stress. The results also showed that glycolysis was inhibited in roots under alkali stress (Table 2).

## Discussion

### Growth, Photosynthesis, and Pigment contents

In the seedling stage, plants are sensitive to adverse external factors, including abiotic stress [29]. *RGR* reflects the condition of the plant and is thus considered as a useful index in determining the degree of stress of a plant. In general, salinity inhibits growth and can even lead to plant death [1]. However, our results showed that *RGR* decreased under salinity stress, with the extent of the decrease under alkali stress being clearly greater than that under salt stress. This indicates that salt stress and alkali stress are distinct types of stress and that the injurious effects of alkali stress on plants are more severe than those of salt stress. The injurious effect of salt stress is commonly caused by low-water potential and ion toxicity; by contrast, alkali stress involves high-pH stress, in addition to these two stress factors [12, 30]. High pH leads to lack of protons and the destruction or inhibition of transmembrane electrochemical potential gradients in cells. The effects of salt stress and alkali stress on photosynthetic activity led us to investigate the mechanisms involved in more detail by examining photosynthesis and pigment contents as indices of stress, since this could provide insights into the nature of the stress-induced damage to the photosynthetic apparatus [12]. In general, *P*_N_ of a plant usually decreases under stress. In the present study, *P*_N_ of wheat was not low under salt stress but decreased sharply under alkali stress. This result implied that salt stress and alkali stress were two distinct stress conditions; the resistance of wheat to salt stress was stronger than that to alkali stress. *g*_s_ was closely correlated with the change in environment water potential. The responses of *g*s in wheat to salinity stress indicate that the change in *g*_s_ could respond to the decrease in environment water potential and intracellular *WC*. The decrease in *g*_s_ might cause the obvious decreases of *E* and *C*_i_ under alkali stress [31]. Chl and Car are the main photosynthetic pigments of higher plants. The contents of Chl and Car was stimulated under salt stress, but decreased sharply under alkali stress. This implies that alkali stress caused Mg precipitation and led to inhibition of Chl synthesis, and it might enhance the activity of the Chl-degrading enzyme chlorophyllase [30, 32]. These results agree with those obtained by Guo et al. and Yang et al. [12, 30, 33, 34].

### Metal elements and free ions

In higher plants, the cytoplasm usually contains low Na^+^ concentrations and high K^+^ concentrations to maintain the function of essential enzyme processes; this state is closely maintained during osmotic adjustment [1, 24]. The results indicated that Na^+^ uptake competes with K^+^ uptake under salt and alkali stresses and that K^+^ percentage of total ionic content decreases, whereas Na^+^ percentage increases; the effects are more pronounced under alkali stress than under salt stress [35]. A considerable response of wheat plant to alkali stress is the strong stimulation of accumulation of Na and Ca in roots by alkali compared with accumulation under salt stress. Under salt stress, the Na^+^ metabolism of plants has at least three processes: compartmentalization (at cellular and/or tissue levels), exclusion (from shoots into roots or from roots to the rhizosphere), and transportation (in vasculature) of the ions. Many plant species have a Na^+^ exclusion mechanism that is dependent on a H^+^ gradient across a root cell membrane [36]. In *Arabidopsis*, the salt overly sensitive l (*SOS*1) protein functions in Na^+^ exclusion from root epidermal cells into the rhizosphere. *SOS*1 protein plays an important role in the control of long-distance transport from roots to shoots and contributes to Na^+^ exclusion from roots to soil [36]. This may be the basis of alkali injury. Tolerance of plants to Na^+^ stress relies on Na^+^ compartmentalization at the cellular and tissue levels, Na^+^ exclusion, and control of long-distance transport in vasculatures. In *Arabidopsis*, the Ca^2+^-responsive AtSOS3–AtSOS2 (AtCIPK24–AtCBL4) protein kinase pathway mediates regulation of the expression and activities of Na^+^ transporters, such as AtSOS1 and AtNHX, which is a Na^+^/H^+^ exchanger that mediates Na^+^ compartmentalization into vacuoles, with Ca^2+^ being the key signal component in the SOS system [24] In summary, on the basis of the above data, we can think that Ca^2+^ plays an important role in plant alkali tolerance to exclude Na. In the present study, salt stress reduces Ca accumulation in wheat roots, but alkali stress strongly enhances its accumulation in roots. Under alkali stress, increasing Ca concentration can immediately trigger SOS–Na excluding system and reduce Na injury to shoots. Plants usually accumulate inorganic anions such as Cl^−^, NO_3_^−^, and SO_4_^2−^ to maintain Na^+^ levels [1, 13]. Cl^−^ levels increased sharply in wheat under salt stress to balance the massive influx of Na^+^, which also caused a severe loss of inorganic negative charges because of the decreased levels of NO_3_^−^ and H_2_PO_4_^−^.

### Metabolic responses to salt and alkali stresses in wheat seedlings

**Fig. 5:**
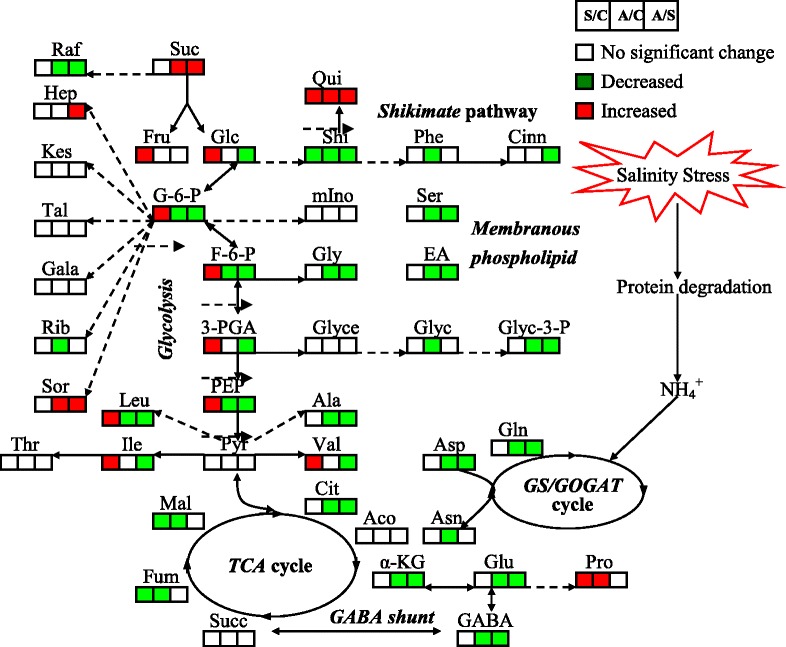
Change in metabolites of the metabolic pathways in leaves of wheat seedlings after 15 d of alkali stress treatment. Proposed metabolic network changes in wheat seedlings subjected to alkali stress, as obtained through OPLS-DA. The metabolites with red boxes denote significant increases; the metabolites with green boxes denote significant decreases (*p* < 0.05). Salt stress/No salinity stress (S/C); Alkali stress/No salinity stress (A/C); Alkali stress/Salt stress (A/S)

**Fig. 6:**
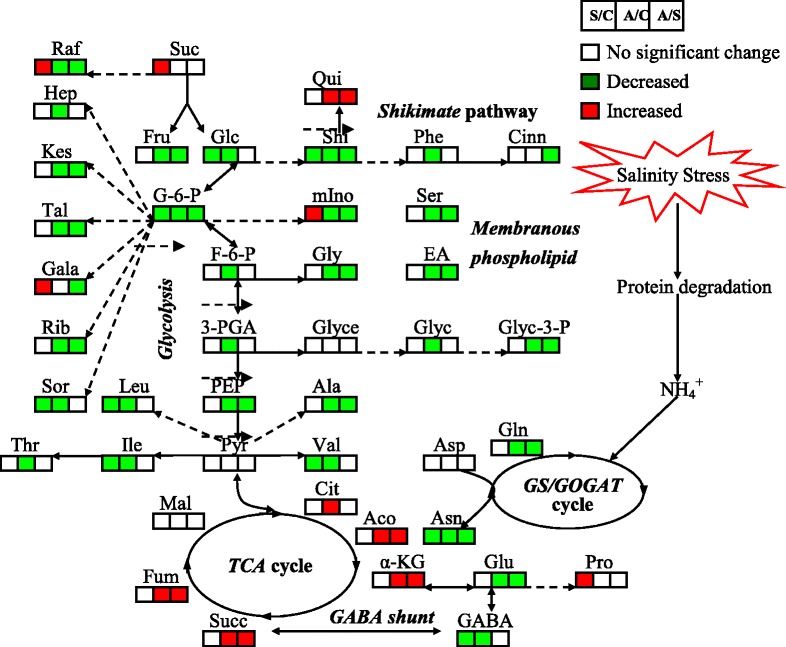
Change in metabolites of the metabolic pathways in roots of wheat seedlings after 15 d of alkali stress treatment. Proposed metabolic network changes in wheat seedlings subjected to alkali stress, as obtained through OPLS-DA. The metabolites with red boxes denote significant increases; the metabolites with green boxes denote significant decreases (*p* < 0.05). Salt stress/No salinity stress (S/C); Alkali stress/No salinity stress (A/C); Alkali stress/Salt stress (A/S)

Osmotic stress and excessive Na^+^ affect root functions when plants are subjected to salinity stress, which induces generation of reactive oxygen species (ROS), such as O_2_^−^, and causes intracellular hyperammonia [37]. Proline is known to play an important role in higher plants in response to osmotic and salinity stress by protecting plant cell membranes and proteins and functioning as a ROS scavenger [38]. In this study, proline accumulated in the cytoplasm of wheat seedlings in response to salinity stress, helping in balancing the osmotic pressure in the vacuoles in response to influx of Na^+^. Proline increase was accompanied with a significant decrease in levels of transamination-related metabolites Glu and GABA, implying that salinity stress promotes Glu conversion into proline with Δ^1^-pyrroline-5-carboxylate synthetase (Figs. 5 and 6). Many studies have shown that the accumulation of some sugars can counter the effects of increased salinity by increasing the osmotic potential to avoid stress [39]. Our results showed that the sugar contents of the wheat seedlings increased in response to salt stress; however, a dramatic decrease in sugar levels was found under alkali stress. In plant cells, sugars were derived from photosynthesis, gluconeogenesis, and degradation of polysaccharides [5, 39]. Since the apparent photosynthetic rate was reported to be similar under control and salt stress, this implies that gluconeogenesis was probably promoted under salt stress to maintain osmotic balance, as well as for sugars to function as carbon storage. The severe negative effect of alkali stress on sugar synthesis and storage may reflect the toxic levels of Na^+^ accumulating in plant cells in a high-pH environment, implying ROS detoxification capacity had been setback by high pH. Our data suggest that sugar accumulation is not a simple passive response to osmotic stress, but rather a result of active metabolic regulation after sensing high pH levels and corresponding alkali stress indicators.

Metabolite responses to salt stress and alkali stress differ. In this study, glycolysis and amino acid synthesis in leaves were likely enhanced under salt stress; by contrast, these processes were inhibited under alkali stress (Figs. 5 and 6). Furthermore, the TCA cycle was inhibited in leaves under salt and alkali stress (Figs. 5 and 6). The results suggest that active synthesis is a basic response of leaves to tolerate salt stress, and energy is not important for leaves to develop salinity stress. Furthermore, an increase in Val, Ile, and Leu was probably associated with inhibited protein biosynthesis or enhanced protein degradation because wheat seedling growth was inhibited under prolonged salt stress; Val, Ile, and Leu are all precursors in the biosynthesis of polyphenols, which function as plant endogenous antioxidants (Figs. 1a and 5). Under alkali stress, the photosynthetic rate decreased significantly, causing reduced production of reducing force and limited N metabolism, which in turn reduces the production of amino acids and inhibits glycolysis (Figs. 5 and 6). The decrease in photosynthetic rate may also affect photorespiration and induce the decrease in photorespiration rate. Under salt stress, TCA cycle and organic acid accumulation were enhanced, but glycolysis and amino acid synthesis were inhibited in roots under alkali stress (Fig. 6). The results indicate that energy and high level of organic acids may be the key adaptive mechanism by which wheat seedlings maintain their intracellular ion balance under alkali stress (Figs. 5 and 6). Organic acids could play a central role in the regulation of intracellular pH by accumulating in vacuoles to neutralize excess cations [13, 32, 34]. Either or both Na^+^ and NO_3_^−^ ions may trigger a signal transduction cascade culminating in the stimulation of organic acid anion synthesis because the main factors contributing to the negative charge deficit in wheat seedlings were excess Na^+^ and limited NO_3_^−^; as a result, various organic acids accumulated. In wheat roots, decrease in amino acids caused by alkali stress may be attributed to limitation of nitrogen metabolism by alkali stress (Fig. 6). Plant roots absorb nitrate (NO_3_^−^), ammonium (NH_4_^+^), and other nutrients from soil using a variety of transporters [40]. For example, AMT protein family members transport NH_4_^+^, whereas NRT protein family members transport nitrate. NH_4_^+^ and NO_3_^−^ uptake mediated by AMT and NRT possibly relies on transmembrane proton gradient [41]. Under alkali stress, the lack of external protons may weaken AMT and NRT activities on the root plasma membrane; thus, NH_4_^+^ and NO_3_^−^ uptake is possibly reduced. Indeed, we observed that alkali stress decreased NO_3_^−^ content in wheat roots to almost zero (Table 1). NO_3_^−^ is reduced to nitrite by nitrate reductase (NR) and then to NH_4_^+^ by nitrite reductase (NiR). NH_4_^+^ from both nitrate reduction and soil are incorporated into organic molecules by glutamine synthetase (GS) and glutamate synthase (Fd-GOGAT and NADH-GOGAT) or through the alternative glutamate dehydrogenase (GDH) pathway [40]. Lack of NO_3_^−^ in wheat roots can decrease NR, GS, and glutamate synthase activities because these enzymes are induced by NO_3_^−^, which should directly limit the synthesis of amino acids. This may affect almost all metabolic processes including the processes observed in our metabolic data.

**Table 1:** Relative concentration and fold changes of major metabolites in leaves of wheat seedlings after 15 d of salt and alkali treatments

Metabolic pathways	Metabolites	Relative concentration			Fold changes		
		CK	SS	AS	log_2_^(SS/CK)^	log_2_^(AS/CK)^	log_2_^(AS/SS)^
*TCA* cycle	Cit	29.28	22.00	6.81	−0.41	−2.10^a^	−1.69^a^
	Aco	1.25	1.02	0.88	−0.30	−0.51	−0.21
	α-KG	0.55	0.52	0.26	−0.09	−1.09^a^	−1.00^a^
	Succ	9.17	6.45	5.28	−0.51	−0.79	−0.29
	Fum	3.70	1.65	1.02	−1.16^a^	−1.86^a^	−0.70
	Mal	19.01	8.10	5.86	−1.23^a^	−1.70^a^	−0.47
*Glycolysis*	Glc	5.66	71.66	9.08	3.66^a^	0.68	−2.98^a^
	G6P	0.56	1.29	0.27	1.19^a^	−1.06^a^	−2.25^a^
	F6P	0.54	1.32	0.23	1.30^a^	−1.24^a^	−2.54^a^
	3-PGA	0.35	0.92	0.31	1.38^a^	−0.18	−1.56^a^
	Pyr	0.68	0.63	0.57	−0.10	−0.25	−0.15
	PEP	0.15	0.62	0.07	2.05^a^	−1.15^a^	−3.19^a^
Amino acids	Glu	270.41	220.74	103.83	−0.29	−1.38^a^	−1.09^a^
	GABA	101.70	85.20	42.42	−0.26	−1.26^a^	−1.01^a^
	Ala	67.72	69.28	24.26	0.03	−1.48^a^	−1.51^a^
	Asp	51.33	32.36	11.90	−0.67	−2.11^a^	−1.44^a^
	Gly	21.96	13.17	4.47	−0.74	−2.30^a^	−1.56^a^
	Thr	9.54	7.76	7.12	−0.30	−0.42	−0.12
	Ser	8.58	9.42	2.56	0.13	−1.74	−1.88
	Val	2.92	5.79	2.90	0.99^a^	−0.01	−1.00^a^
	Pro	0.39	1.31	1.29	1.73^a^	1.72^a^	−0.02
	Phe	0.51	0.42	0.24	−0.29	−1.10^a^	−0.82
	Gln	0.60	0.37	0.09	−0.68	−2.72^a^	−2.04^a^
	Ile	0.42	2.89	0.48	2.77^a^	0.19	−2.58^a^
	Leu	0.07	0.42	0.03	2.66^a^	−1.02^a^	−3.68^a^
	Asn	0.06	0.05	0.03	−0.17	−1.12^a^	−0.96
Sugars and polyols	Fru	162.82	327.02	224.24	1.01^a^	0.46	−0.54
	Suc	0.54	0.92	1.72	0.76	1.67^a^	0.90^a^
	Tal	29.78	25.25	22.97	−0.24	−0.37	−0.14
	Kes	0.29	0.40	0.33	0.45	0.19	−0.26
	Hep	0.56	0.36	0.85	−0.64	0.60	1.24^a^
	mIno	20.79	18.76	15.98	−0.15	−0.38	−0.23
	Sor	0.52	0.39	2.35	−0.42	2.16^a^	2.58^a^
	Rib	30.99	19.85	14.86	−0.64	−1.06^a^	−0.42
	Gala	23.45	18.76	16.26	−0.32	−0.53	−0.21
	Raf	14.24	13.45	4.35	−0.08	−1.71^a^	−1.63^a^

The relative concentration of each metabolite is an average of data from six biological replicates obtained through GC–MS. Fold changes were calculated using the formula log_2_^(treatment/control)^

^a^indicates significance (*p* < 0.05)

**Table 2:** Relative concentration and fold changes of major metabolites in roots of wheat seedlings after 15 d of salt and alkali treatments

Metabolic pathways	Metabolites	Relative concentration			Fold changes		
		CK	SS	AS	log_2_^(SS/CK)^	log_2_^(AS/CK)^	log_2_^(AS/SS)^
*TCA* cycle	Cit	70.05	114.56	166.40	0.71	1.25^a^	0.54
	Aco	1.37	1.44	2.87	0.07	1.07^a^	1.00^a^
	α-KG	0.15	0.27	0.59	0.81	1.94^a^	1.13^a^
	Succ	17.80	11.37	86.02	−0.65	2.27^a^	2.92^a^
	Fum	1.25	1.04	7.21	−0.27	2.53^a^	2.80^a^
	Mal	10.44	16.43	18.29	0.65	0.81	0.15
*Glycolysis*	Glc	22.49	11.19	9.45	−1.01^a^	−1.25^a^	−0.24
	G6P	0.49	0.23	0.10	−1.09^a^	−2.32^a^	−1.23^a^
	F6P	0.19	0.10	0.05	−0.95	−1.96^a^	−1.01^a^
	3-PGA	0.44	0.27	0.14	−0.72	−1.63^a^	−0.92
	Pyr	0.49	0.52	0.39	0.09	−0.34	−0.43
	PEP	0.64	0.61	0.25	−0.07	−1.34^a^	−1.27^a^
Amino acids	Glu	134.99	141.95	36.04	0.07	−1.91^a^	−1.98^a^
	GABA	104.32	46.87	33.20	−1.15^a^	−1.65^a^	−0.50
	Ala	151.29	98.92	14.73	−0.61	−3.36^a^	−2.75^a^
	Asp	12.33	7.44	7.28	−0.73	−0.76	−0.03
	Gly	7.45	4.09	0.97	−0.87	−2.94^a^	−2.07^a^
	Thr	9.71	4.95	1.50	−0.97	−2.70^a^	−1.7^a^2
	Ser	16.05	11.41	2.22	−0.49	−2.85^a^	−2.36^a^
	Val	17.42	8.19	5.28	−1.09^a^	−1.72^a^	−0.64
	Pro	8.54	17.36	14.58	1.02^a^	0.77	−0.25
	Phe	0.41	0.19	0.16	−1.08^a^	−1.31^a^	−0.23
	Gln	0.11	0.12	0.02	0.17	−2.20^a^	−2.36 ^a^
	Ile	7.50	3.06	2.51	−1.29^a^	−1.58^a^	−0.29
	Leu	0.63	0.19	0.14	−1.73^a^	−2.18^a^	−0.44
	Asn	16.66	3.07	1.29	−2.44^a^	−3.69^a^	−1.25^a^
Sugars and polyols	Fru	860.33	684.72	237.87	−0.33	−1.85^a^	−1.53^a^
	Suc	96.20	180.38	104.07	0.91	0.11	−0.79
	Tal	119.04	85.27	26.98	−0.48	−2.14^a^	−1.66^a^
	Kes	60.63	84.66	24.67	0.48	−1.30^a^	−1.78^a^
	Hep	0.80	0.29	0.18	−1.47^a^	−2.19^a^	−0.72
	mIno	11.07	24.44	4.62	1.14^a^	−1.26^a^	−2.40^a^
	Sor	9.78	3.13	3.42	−1.65^a^	−1.52^a^	0.13
	Rib	9.02	5.24	1.56	−0.78	−2.53^a^	−1.75^a^
	Gala	0.85	3.22	0.84	1.92^a^	−0.02	−1.94^a^
	Raf	0.39	1.01	0.10	1.39^a^	−1.91^a^	−3.29^a^

The relative concentration of each metabolite is an average of data from six biological replicates obtained through GC–MS. Fold changes were calculated using the formula log_2_^(treatment/control)^

^a^indicates significance (*p* < 0.05)

## Conclusions

This paper indicated that salt and alkali stresses are two different stresses, the inhibitory effects of alkali stress on wheat growth and photosynthesis were greater than those of salt stress. Alkali stress induced the production of high amounts of phosphate and metal ion precipitate, except Na and K; as a consequence, ionic activities and free concentrations of various ions decreased. Salt stress reduced Ca accumulation in wheat roots; by contrast, alkali stress strongly induced Ca accumulation in roots. Under alkali stress, increasing Ca concentration can immediately trigger *SOS*1 Na exclusion system and reduce Na injury. We detected the 75 metabolites that were different among the treatments, including organic acids, amino acids, and carbohydrates. A dynamic trajectory was observed in wheat metabolic profiles in response to salt and alkali stress. Based on the comparison results of metabolic profiles and SPAD values between salt stress and alkali stress, the harmful effects of alkali stress on the distribution and accumulation of metabolites were significantly greater than those of salt stress; these outcomes correspond to specific detrimental effects of a highly alkaline environment. Prolonged salt stress induced progressive accumulation of sugars by gluconeogenesis to avoid osmotic stress. Such treatment also promoted energy metabolism in roots and active synthesis in leaves to develop salt tolerance in wheat. Alkali stress (at high pH) significantly inhibited photosynthetic rate; thus, sugar production was reduced, N metabolism was limited, amino acid production was reduced, and glycolysis was inhibited. Alkali stress also severely inhibited nitrate uptake by roots and subsequent N assimilation, possibly decreasing the accumulation of amino acids. Therefore, energy and high organic acid concentrations are possible key adaptive factors needed by wheat to maintain intracellular ion balance and regulate high pH under alkali stress.

## Additional files

Additional file 1:
**Relative concentration and fold changes of 75 metabolites in leaves of wheat seedlings after 15 days of salt and alkali treatment.** The relative concentration of each metabolite is an average of data from five biological replicates using GC-MS. The fold changes was calculated using the formula log_2_^(treatment/control)^. *indicate significant (*P* < 0.05). Additional file 2:
**Relative concentration and fold changes of 75 metabolites in roots of wheat seedlings after 15 days of salt and alkali treatment.** The relative concentration of each metabolite is an average of data from five biological replicates using GC-MS. The fold changes was calculated using the formula log_2_^(treatment/control)^. *indicate significant (*P* < 0.05).

## Abbreviations

Cit: Citric acid
Aco: Aconitic Acid
α-KG: α-ketoglutaric acid
Succ: Succinic acid
Fum: Fumaric acid
Mal: Malic acid
Glc: Glucose
G6P: Glucose-6-phosphate
F6P: Fructose-6-phosphate
3-PGA: 3-phosphoglycerate
Pyr: Pyruvate
PEP: Phosphoenolpyruvate
Glu: Glutamate
GABA: γ-aminobutyric acid
Ala: Alanine
Asp: Aspartic acid
Gly: Glycine
Thr: Threonine
Ser: Serine
Val: Valine
Pro: Proline
Phe: Phenylalanine
Gln: Glutamine
Ile: Isoleucine
Leu: Leucine
Asn: Asparagine
Fru: Fructose
Suc: Sucrose
Tal: Talose
Kes: Kestose
Hep: Heptulose
mIno: myo-Inositol
Sor: Sorbitol
Rib: Ribose
Gala: Galactinol
Raf: Raffinose
