# Supplementary material for: Comparative metabolic responses and adaptive strategies of wheat (Triticum aestivum) to salt and alkali stress
Source: BMC Plant Biol. 2015 Jul 7;15:170. doi: 10.1186/s12870-015-0546-x (PMC4492011; doi:10.1186/s12870-015-0546-x)
Supplement: Additional file 1: — Relative concentration and fold changes of 75 metabolites in leaves of wheat seedlings after 15 days of salt and alkali treatment. The relative concentration of each metabolite is an average of data from five biological replicates using GC-MS. The fold changes was calculated using the formula log2 (treatment/control). *indicate significant (P < 0.05). [file 12870_2015_546_MOESM1_ESM.doc]

| Metabolic  pathways | Metabolites name | Relative concentration | | | Fold changes | | |
| --- | --- | --- | --- | --- | --- | --- | --- |
|  |  | CK | SS | AS | Log_2_^(SS/CK)^ | Log_2_^(AS/CK)^ | Log_2_^(AS/SS)^ |
| **TCA cycle** | Citric acid | 29.28 | 22.00 | 6.81 | -0.41 | -2.10^﹡^ | -1.69^﹡^ |
|  | Aconitic Acid | 1.25 | 1.02 | 0.88 | -0.30 | -0.51 | -0.21 |
|  | α-ketoglutaric acid | 0.55 | 0.52 | 0.26 | -0.09 | -1.09^﹡^ | -1.00^﹡^ |
|  | Succinic acid | 9.17 | 6.45 | 5.28 | -0.51 | -0.79 | -0.29 |
|  | Fumaric acid | 3.70 | 1.65 | 1.02 | -1.16^﹡^ | -1.86^﹡^ | -0.70 |
|  | Malic acid | 19.01 | 8.10 | 5.86 | -1.23^﹡^ | -1.70^﹡^ | -0.47 |
| **Glycolysis** | Glucose | 5.66 | 71.66 | 9.08 | 3.66^﹡^ | 0.68 | -2.98^﹡^ |
|  | Glucose-6-phosphate | 0.56 | 1.29 | 0.27 | 1.19^﹡^ | -1.06^﹡^ | -2.25^﹡^ |
|  | Fructose-6-phosphate | 0.54 | 1.32 | 0.23 | 1.30^﹡^ | -1.24^﹡^ | -2.54^﹡^ |
|  | 3-phosphoglycerate | 0.35 | 0.92 | 0.31 | 1.38^﹡^ | -0.18 | -1.56^﹡^ |
|  | Pyruvate | 0.68 | 0.63 | 0.57 | -0.10 | -0.25 | -0.15 |
|  | Phosphoenolpyruvate | 0.15 | 0.62 | 0.07 | 2.05^﹡^ | -1.15^﹡^ | -3.19^﹡^ |
| **Amino acids** | Glutamate | 270.41 | 220.74 | 103.83 | -0.29 | -1.38^﹡^ | -1.09 |
|  | Alanine | 67.72 | 69.28 | 24.26 | 0.03 | -1.48^﹡^ | -1.51 |
|  | γ-aminobutyric acid | 101.70 | 85.20 | 42.42 | -0.26 | -1.26^﹡^ | -1.01 |
|  | Valine | 2.92 | 5.79 | 2.90 | 0.99^﹡^ | -0.01 | -1.00 |
|  | Asparagine | 0.06 | 0.05 | 0.03 | -0.17 | -1.12^﹡^ | -0.96 |
|  | Serine | 8.58 | 9.42 | 2.56 | 0.13 | -1.74^﹡^ | -1.88 |
|  | Aspartic acid | 51.33 | 32.36 | 11.90 | -0.67 | -2.11^﹡^ | -1.44 |
|  | Threonine | 9.54 | 7.76 | 7.12 | -0.30 | -0.42 | -0.12 |
|  | Proline | 0.39 | 1.31 | 1.29 | 1.73^﹡^ | 1.72^﹡^ | -0.02 |
|  | Isoleucine | 0.42 | 2.89 | 0.48 | 2.77^﹡^ | 0.19 | -2.58 |
|  | Glycine | 21.96 | 13.17 | 4.47 | -0.74 | -2.30^﹡^ | -1.56 |
|  | Lysine | 0.14 | 0.20 | 1.34 | 0.44 | 3.22^﹡^ | 2.78 |
|  | leucine | 0.07 | 0.42 | 0.03 | 2.66^﹡^ | -1.02^﹡^ | -3.68 |
|  | Phenylalanine | 0.51 | 0.42 | 0.24 | -0.29 | -1.10^﹡^ | -0.82 |
|  | Tyrosine | 0.11 | 0.13 | 0.05 | 0.31 | -1.04^﹡^ | -1.35 |
|  | Glutamine | 0.60 | 0.37 | 0.09 | -0.68 | -2.72^﹡^ | -2.04 |
| **Sugars and polyols** | Fructose | 162.82 | 327.02 | 224.24 | 1.01^﹡^ | 0.46 | -0.54 |
|  | Sucrose | 0.54 | 0.92 | 1.72 | 0.76 | 1.67^﹡^ | 0.90 |
|  | Talose | 29.78 | 25.25 | 22.97 | -0.24 | -0.37 | -0.14 |
|  | Kestose | 0.29 | 0.40 | 0.33 | 0.45 | 0.19 | -0.26 |
|  | Heptulose | 0.56 | 0.36 | 0.85 | -0.64 | 0.60 | 1.24 |
|  | myo-Inositol | 20.79 | 18.76 | 15.98 | -0.15 | -0.38 | -0.23 |
|  | Sorbitol | 0.52 | 0.39 | 2.35 | -0.42 | 2.16^﹡^ | 2.58 |
|  | Ribose | 30.99 | 19.85 | 14.86 | -0.64 | -1.06^﹡^ | -0.42 |
|  | Glucose-1-phosphate | 0.50 | 0.38 | 0.42 | -0.41 | -0.25 | 0.16 |
|  | Xylose | 1.44 | 1.01 | 2.01 | -0.51 | 0.48 | 0.99 |
|  | Xylitol | 0.39 | 0.22 | 0.16 | -0.86 | -1.28^﹡^ | -0.42 |
|  | Trehalose | 0.08 | 0.36 | 0.39 | 2.22^﹡^ | 2.32^﹡^ | 0.10 |
|  | Sophorose | 0.19 | 0.11 | 0.06 | -0.72 | -1.52^﹡^ | -0.81 |
|  | Lyxose | 0.79 | 0.44 | 1.65 | -0.83 | 1.06^﹡^ | 1.90 |
|  | Lanosterol | 2.05 | 1.74 | 1.19 | -0.23 | -0.78 | -0.55 |
|  | Galactinol | 23.45 | 18.76 | 16.26 | -0.32 | -0.53 | -0.21 |
|  | Raffinose | 14.24 | 13.45 | 4.35 | -0.08 | -1.71^﹡^ | -1.63 |
|  | Maltose | 0.39 | 0.17 | 0.08 | -1.15^﹡^ | -2.24^﹡^ | -1.09 |
|  | Phytol | 3.71 | 3.40 | 2.77 | -0.13 | -0.42 | -0.29 |
|  | Gentiobiose | 0.26 | 0.14 | 0.63 | -0.91 | 1.30^﹡^ | 2.21 |
|  | Galactose | 0.18 | 0.12 | 0.04 | -0.57 | -2.11^﹡^ | -1.53 |
|  | Lactose | 0.35 | 0.27 | 0.51 | -0.41 | 0.52 | 0.93 |
| **Organic acids** | Shikimic acid | 68.78 | 33.37 | 13.96 | -1.04^﹡^ | -2.30^﹡^ | -1.26 |
|  | Itaconic acid | 59.95 | 48.23 | 48.29 | -0.31 | -0.31 | 0.00 |
|  | Quinic acid | 18.02 | 7.95 | 2.74 | -1.18^﹡^ | -2.72^﹡^ | -1.54 |
|  | Palmitic acid | 13.81 | 14.09 | 7.27 | 0.03 | -0.93 | -0.96 |
|  | Ascorbic Acid | 6.33 | 6.20 | 3.27 | -0.03 | -0.95 | -0.93 |
|  | Glyceric acid | 4.27 | 3.08 | 3.51 | -0.47 | -0.28 | 0.19 |
|  | Stearic acid | 3.56 | 3.19 | 1.86 | -0.16 | -0.94 | -0.78 |
|  | Glycolic acid | 2.98 | 2.44 | 1.46 | -0.29 | -1.03^﹡^ | -0.75 |
|  | Threonic acid | 2.38 | 1.57 | 1.64 | -0.61 | -0.54 | 0.07 |
|  | Linolenic acid | 2.04 | 3.65 | 1.14 | 0.84 | -0.84 | -1.68 |
|  | Lactic acid | 1.45 | 1.47 | 1.13 | 0.03 | -0.36 | -0.39 |
|  | Linoleic acid | 0.12 | 0.11 | 0.05 | -0.09 | -1.21^﹡^ | -1.12 |
|  | Benzoic acid | 0.43 | 0.31 | 0.36 | -0.47 | -0.24 | 0.22 |
|  | Oleic acid | 0.02 | 0.03 | 0.01 | 0.40 | -1.54^﹡^ | -1.94 |
|  | Arachidic acid | 0.44 | 0.47 | 0.15 | 0.08 | -1.54^﹡^ | -1.62 |
|  | Malonic acid | 2.25 | 1.63 | 1.47 | -0.47 | -0.61 | -0.15 |
|  | Valeric acid | 0.12 | 0.06 | 0.05 | -0.94 | -1.24^﹡^ | -0.30 |
|  | Cinnamic acid | 0.81 | 0.91 | 0.47 | 0.17 | -0.77 | -0.94 |
| **Others** | Phosphate | 33.57 | 44.16 | 11.06 | 0.40 | -1.60^﹡^ | -2.00 |
|  | Glycerol | 20.65 | 13.89 | 9.62 | -0.57 | -1.10^﹡^ | -0.53 |
|  | Diglycerol | 1.51 | 1.38 | 1.90 | -0.13 | 0.33 | 0.46 |
|  | Glycerol-3-phosphate | 12.56 | 10.89 | 4.74 | -0.21 | -1.41^﹡^ | -1.20 |
|  | Pyridine | 19.92 | 14.12 | 9.71 | -0.50 | -1.04^﹡^ | -0.54 |
|  | Ethanolamine | 4.15 | 5.59 | 4.95 | 0.43 | 0.25 | -0.18 |
|  | Propane | 1.01 | 1.09 | 0.79 | 0.10 | -0.35 | -0.45 |
